# Unveiling cryptic species diversity of flowering plants: successful biological species identification of Asian *Mitella *using nuclear ribosomal DNA sequences

**DOI:** 10.1186/1471-2148-9-105

**Published:** 2009-05-16

**Authors:** Yudai Okuyama, Makoto Kato

**Affiliations:** 1 Graduate School of Human and Environmental Studies, Kyoto University, Sakyo, Kyoto, 606-8501, Japan; 2 Current address: Tsukuba Botanical Garden, National Museum of Nature and Science, 4-1-1 Amakubo, Tsukuba 305-0005, Japan

## Abstract

**Background:**

Although DNA sequence analysis is becoming a powerful tool for identifying species, it is not easy to assess whether the observed genetic disparity corresponds to reproductive isolation. Here, we compared the efficiency of biological species identification between nuclear ribosomal and chloroplast DNA sequences, focusing on an Asian endemic perennial lineage of *Mitella *(*Asimitellaria*; Saxifragaceae). We performed artificial cross experiments for 43 pairs of ten taxonomic species, and examined their F1 hybrid pollen fertility in vitro as a quantitative measure of postzygotic reproductive isolation.

**Results:**

A nonlinear, multiple regression analysis indicated that the nuclear ribosomal DNA distances are sufficient to explain the observed pattern of F1 hybrid pollen fertility, and supplementation with chloroplast DNA distance data does not improve the explanatory power. Overall, with the exception of a recently diverged species complex with more than three biological species, nuclear ribosomal DNA sequences successfully circumscribed ten distinct biological species, of which two have not been described (and an additional one has not been regarded as a distinct taxonomic species) to date.

**Conclusion:**

We propose that nuclear ribosomal DNA sequences contribute to reliable identification of reproductively isolated and cryptic species of *Mitella*. More comparable studies for other plant groups are needed to generalize our findings to flowering plants.

## Background

Plant systematics is one of the most active areas of biology because of marked progress in molecular phylogenetics during recent decades [1]. Many of the long-standing enigmas regarding systematic positions of various taxonomic groups, for example, the relationships among gymnosperms, basal angiosperms, monocots, and dicots, have been resolved, and overall agreement has now been reached regarding circumscription of the major orders and families, with current practical taxonomic systems now following the Angiosperm Phylogeny Group (APG) system [2,3] with little controversy. The number of research articles on plant molecular phylogenetics has increased markedly in recent years, focusing mainly on extending studies to lower taxonomic groups. At the same time, the rapid accumulation of DNA sequence data for phylogenetic studies has prompted recent endeavors to use them for precise and efficient delineation of biodiversity (DNA taxonomy [4,5]).

Many studies attempting to resolve plant evolutionary relationships and/or to identify plant species using DNA sequences have assumed that intraspecific genetic diversity is usually lower than interspecific genetic diversity and that sequences derived from a species usually form a monophyletic group. Consequently, sampling of a few individuals (or even only one) is considered sufficient to represent the genetic characteristic of the species. However, these assumptions are not thoroughly supported by empirical data. For example, a recent survey of DNA sequences of a nuclear-encoded gene in the *Pinus *subgenus *Strobus *indicated that 58% of the taxonomic species studied did not form a monophyletic group [6]. These authors also reported that many published studies that include multiple accessions per taxonomic species failed to reconstruct species monophyly for up to 100% of the species examined. If such species non-monophyly is common among plants, any attempt at DNA-based approaches for taxonomy would lose their relevance. Moreover, the frequency of allelic non-monophyly among plant biological species is not only methodologically but also conceptually crucial for our understanding of plant speciation. Assuming that long-term maintenance of reproductively distinct species results in allelic uniqueness of some, if not all, gene loci for each species, this should directly lead to a classic debate on the nature of plant species [7-10], because a species that cannot be recognized genetically may not be a real entity (but see [11]). Nevertheless, very little information is available regarding whether a plant species can indeed be regularly recognized as a genetically distinct group (e.g., only 17 studies are available [6]). More specifically, most of these studies examining the correspondence between supported clades in a phylogenetic tree and species rely heavily on traditional taxonomic species circumscriptions, obscuring whether such patterns of species non-monophyly, if observed, can be attributed to true non-monophyly or only to poor resolution of the present taxonomic system.

A more constructive approach to establish methodologies for plant DNA taxonomy would be to find genetic markers that are most likely to achieve species monophyly of the group under study, because the probability of supporting species monophyly should vary across markers and lineages in response to the marker-specific coalescence time and lineage-specific life history traits. Importantly, although many recent papers on plant DNA barcoding have placed strong emphasis on the use of markers on the chloroplast genome [12-15], the chloroplast genome constitutes a non-recombining, single linkage group so that the differences among markers on the chloroplast genome might be limited to differences in the amount of information or its resolution, but not to their accuracy.

Assuming that biological species are the entities that have some, if not a complete, degree of reproductive isolation from each other, such an ideal marker for species delimitation should also have the capacity to estimate the degree of reproductive isolation among the plant individuals, from which sequence data are available but species identities are unknown. Nevertheless, few studies have compared the relationship between genetic divergence and reproductive isolation in plants. To our knowledge, only three empirical studies (genus *Glycine *[Fabaceae], *Silene *[Caryophyllaceae], and *Streptanthus *[Brassicaceae]) have been published in which a general trend of correlation between pre-/postzygotic reproductive isolation and genetic distance was observed, and each of these studies used only one measure of genetic distance (nuclear ribosomal ITS DNA sequences for the former two, and allozyme distance for the last [16]). In fact, no study has compared the relationship of different gene loci to the degree of reproductive isolation.

Here, we report that species within the Asian *Mitella *section *Asimitellaria *can mostly be recognized as a distinct, monophyletic clade that exhibits reproductive isolation (measured by sterility of pollen from F1 hybrids) based on nuclear ribosomal external and internal transcribed spacer (ETS and ITS) DNA sequences. In contrast, we found that the relatively long sequence reads (> 1.5 kbp) of the chloroplast *psbA-trnH *interspecific spacer plus the *matK *gene, which are the most frequently used markers for plant DNA barcoding, were much less effective for recognizing the biological species boundaries likely due to natural hybridizations in *Asimitellaria*.

*Asimitellaria *is a monophyletic group of perennials that diversified into more than ten species exclusively within Japan and Taiwan, which enables comprehensive sampling of genetic diversity that presumably derived from a single ancestor. All *Asimitellaria *species and varieties have the same chromosome number (2n = 28), with very few exceptions of intraspecific variations in chromosome number, that is, some triploid plants of *M. pauciflora *in the northernmost populations [17], implying that a complex polyploid formation has not been responsible for speciation. By analyzing a comprehensive collection of nuclear ribosomal ETS and ITS DNA sequences and the chloroplast *psbA-trnH *spacer and *matK *gene DNA sequences from samples of *Asimitellaria *plants throughout their distribution range, we first examined if distinct genotypic clusters reflect species circumscription. Furthermore, we examined pollen fertility of 43 lines of artificially crossed F1 hybrids to determine whether reproductive isolation occurs between species and how the parental genetic distances are related to the observed degrees of reproductive isolation. Furthermore, we determined whether the distinct cluster recognized by the nucleotide sequence data corresponds to a distinct taxonomic or biological species. Finally, we discuss the utility and limitations of these DNA sequences as identification tools for plant species.

## Methods

### Study organisms

**Figure 1:**
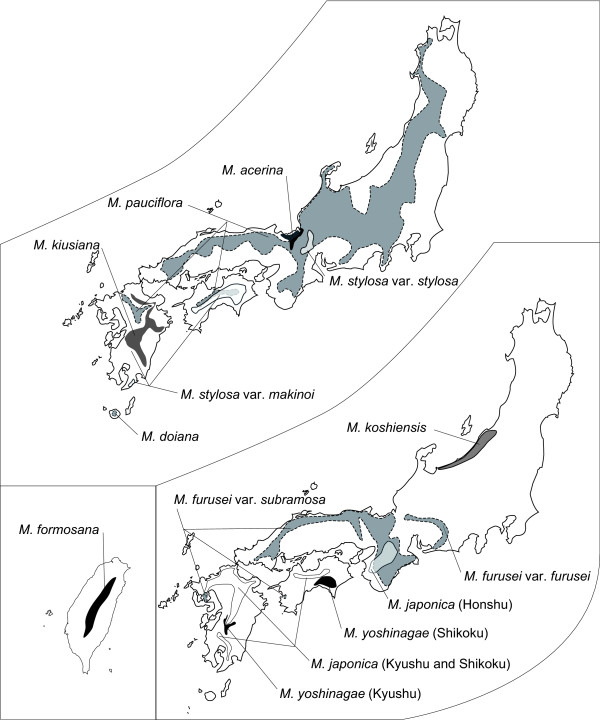
**Geographic distribution ranges of ten *Asimitellaria *taxonomic species (and an additional two taxonomic varieties) drawn from the records of Wakabayashi (1973) and our own studies**. Note that the taxonomic species are arbitrarily separated onto two maps of Japan to minimize overlap.

**Figure 2:**
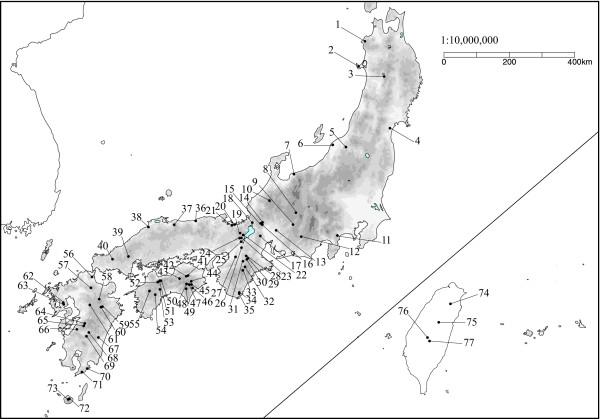
**Locations of 77 populations from which *Asimitellaria *plants were collected**. The population numbers 1–77 correspond to those in Additional file 1: Table S1.

The genus *Mitella *section *Asimitellaria *(Saxifragales; Saxifragaceae in the APG system) is a monophyletic group of perennials endemic to Japan and Taiwan. Nine species and an additional two varieties endemic to Japan and one species endemic to Taiwan have been described to date [18]. We sampled 158 individuals of all ten *Asimitellaria *species and two varieties throughout their distribution range (Figure 1) for DNA sequencing, of which 17 individuals were only sequenced for nuclear ribosomal DNA but not for chloroplast DNA because of sample loss. For each species and variety, sequences from 2 to 20 populations encompassed the entire distribution range (Figure 2; Additional file 1: Table S1). Nine other species of the genus *Mitella *were used as outgroups, as they are clearly not included in *Asimitellaria *[19]. Overall, 105, 150, and 116 individuals were newly sequenced for nuclear ribosomal DNA, chloroplast *psbA-trnH*, and *matK*, respectively, whereas the remaining sequences were obtained from previous studies [19,20].

The plants used in the present study were either cultivated at Tsukuba Botanical Garden, or deposited as voucher specimens in the Kyoto University Herbarium (KYO).

### Analyses of nucleotide sequences

Generally, DNA extraction and sequencing followed methods described elsewhere [19,20]. A detailed description of the method is available in additional file 2. The primer sequences used in the present study are listed in Additional file 1: Table S2. The DNA sequences newly generated in this study were deposited in DDBJ (National Institute of Genetics, Mishima, Japan) under accession nos. AB492287–AB492762. The obtained sequences of nuclear ribosomal ETS and ITS regions and the chloroplast *matK *gene were easily aligned manually, with very few insertions/deletions (indels). In contrast, sequence alignment of the chloroplast *psbA-trnH *spacer was less straightforward. For example, an 8- to 26-base stretch of poly-T sequences with very few other bases on the aligned site 170–195 of the chloroplast *psbA-trnH *spacer was impossible to align and therefore excluded from the data. Moreover, a careful inspection revealed a 37-base inversion on the aligned site 100–136 of the chloroplast *psbA-trnH *spacer. We therefore excluded this region and instead coded it as a single binary character with character states inverted or non-inverted. The indels in the aligned matrix were unambiguously coded as separate characters using the methods described in Simmons and Ochoterena [21]. The 5.8S region that is flanked by the ITS-1 and ITS-2 regions was removed from the dataset, as this region is missing in some of the sequences from previous studies. Genetic distances between all pairs of plant individuals were calculated separately with PAUP*4.0b10 [22] for each of the nuclear and chloroplast DNA datasets, using the Tamura-Nei + I + Γ model of nucleotide substitutions (gamma shape = 0.8578, proportion of invariable sites = 0.3018) for the nuclear dataset, and the K81uf + Γ model (gamma shape = 0.2834) for the chloroplast DNA dataset, both of which were selected using ModelTest 3.7 [23]. The relationship between all pairwise genetic distances calculated from each of nuclear and chloroplast dataset (hereafter referred to as nuclear genetic distance and chloroplast genetic distance, respectively) were also examined. All statistical analyses were performed using the R package version 2.7.0 [24] unless otherwise mentioned.

For cladistic grouping of collected nucleotide sequences, maximum parsimony (MP), neighbor-joining (NJ), and Bayesian (Bayesian inference: BI) tree searches were performed using PAUP*4.0b10 for MP and NJ analyses and MrBayes 3.1 [25] for BI. A heuristic search with tree bisection-reconnection (TBR) branch swapping and 100 additional sequence replicates, saving a maximum of 100 trees per replicate, was used for the MP tree search, and a distance measure under the maximum likelihood settings was used for the NJ tree search. To assess topological uncertainty, bootstrapping (1000 and 10,000 replicates for MP and NJ, respectively) was also performed, using the same settings as in the original tree searches, except that we reduced both the maximum tree number and additional sequence replicates to ten in the MP analysis. For BI, the GTR + I + Γ base substitution model, F81 base substitution model, and GTR+ Γ base substitution model were used with an uninformative prior for combined nuclear ribosomal ETS and ITS, chloroplast *psbA-trnH*, and chloroplast *matK*, respectively. The standard model with equal rate variation among sites was used for indel and inversion data. The nucleotide substitution models for BI were selected using MrModelTest 2.2 [26]. Two independent runs of Markov chain Monte Carlo (MCMC) simulation were allowed for 1.2 or 2.2 million generations each (for nuclear and chloroplast datasets, respectively), with trees sampled every 1000 generations, to achieve independence among samples. The likelihood scores of the obtained trees were plotted to confirm that the two independent runs reached virtually identical stationarity well before the first 201 or 1201 trees of each run, which were discarded as burn-in. As a result, 2000 trees were retained, and a majority-rule consensus tree (hereafter referred to as the Bayesian consensus tree) was constructed using these trees.

### Examination of F1 hybrid fertility via artificial cross experiments

To examine the presence of reproductive isolation among and within the genotypic clusters identified from the ETS and ITS sequence data, we performed artificial cross experiments using 55 individuals of ten *Asimitellaria *species and two varieties collected from 26 populations. Specifically, because we observed genotypic clusters within each of *M. stylosa*, *M. japonica*, and *M. yoshinagae*, we suspected that these clusters may form distinct species, and therefore examined the presence of hybrid sterility among these clusters. We also checked for the presence of hybrid sterility among *M. furusei*, *M. pauciflora*, and *M. koshiensis*, as this species complex could not be separated by genotypic clustering, implying the need for assessment of concordance between taxonomic species and boundaries of biological species within the complex.

We used the pollen germination ratio of F1 hybrids for 43 combinations of crosses (corresponding to cross strain nos. 10–52 listed in Table 1) as a measure of F1 hybrid fertility, because this measure was highly quantitative and highly variable among the cross designs. The other measures, such as fruit/seed set of crossed plants and F1 plant growth were generally high, with a few exceptions in crosses between very distantly related species.

**Table 1:** The parental genetic distances measured with chloroplast and nuclear DNA, and average pollen fertility of nine wild-collected species (strain ID nos. 1–9) and 43 F1 hybrids (strain ID nos. 10–52).

Strain ID	Cross design^a^	Maternal (Population^b^)	Paternal (Population^b^)	N^c^	Nuclear genetic distance	Chloroplast genetic distance	Average fertility (± s.d.)
1	wild, Clade A	*M. pauciflora*		5	0	0	0.872 ± 0.123
2	wild, Clade A	*M. koshiensis*		4	0	0	0.798 ± 0.169
3	wild, Clade A	*M. furusei *var. *subramosa*		5	0	0	0.838 ± 0.107
4	wild, Clade B	*M. kiusiana*		6	0	0	0.685 ± 0.277
5	wild, Clade B	*M. stylosa *var. *stylosa*		7	0	0	0.813 ± 0.101
6	wild, Clade B	*M. stylosa *var. *makinoi*		8	0	0	0.860 ± 0.122
7	wild, Clade C	*M. japonica *(Shikoku & Kyushu)		6	0	0	0.923 ± 0.096
8	wild, Clade C	*M. japonica *(Honshu)		4	0	0	0.808 ± 0.123
9	wild, Clade C	*M. yoshinagae *(Kyushu)		2	0	0	0.995 ± 0.007
10	WS, Clade A	*M. furusei *var.*furusei *(16)	*M. furusei *var.*subramosa *(29)	6	0.0087	0.0033	0.170 ± 0.104
11	WS, Clade A	*M. furusei *var.*subramosa *(29)	*M. furusei *var.*furusei *(16)	4	0.0087	0.0033	0.205 ± 0.205
12	WS, Clade A	*M. furusei *var.*subramosa *(18)	*M. furusei *var.*subramosa *(38)	6	0.0121	0.0061	0.103 ± 0.024
13	WS, Clade A	*M. furusei *var.*subramosa *(38)	*M. furusei *var.*subramosa *(18)	5	0.0121	0.0061	0.028 ± 0.013
14	WS, Clade B	*M. stylosa *var.*stylosa *(15)	*M. stylosa *var. *makinoi *(55)	12	0.0133	0.0033	0.417 ± 0.217
15	WS, Clade C	*M. japonica *(60)	*M. japonica *(60)	3	0	0	0.837 ± 0.015
16	WS, Clade C	*M. japonica *(70)	*M. japonica *(56)	9	0.0023	0.004	0.867 ± 0.065
17	WS, Clade C	*M. japonica *(70)	*M. japonica *(51)	19	0.0023	0.0027	0.788 ± 0.114
18	WS, Clade C	*M. yoshinagae *(44)	*M. yoshinagae *(64)	6	0.0217	0.0007	0.019 ± 0.029
19	WS, Clade C	*M. japonica *(60)	*M. japonica *(29)	9	0.0364	0.0007	0.348 ± 0.348
20	WS, Clade C	*M. japonica *(29)	*M. japonica *(60)	8	0.0364	0.0007	0.260 ± 0.178
21	WS, Clade C	*M. japonica *(70)	*M. japonica *(29)	5	0.0378	0.0027	0.038 ± 0.064
22	WS, Clade C	*M. japonica *(29)	*M. japonica *(56)	9	0.0385	0.0013	0.034 ± 0.023
23	BS, Clade A	*M. furusei *var.*subramosa *(29)	*M. pauciflora *(29)	6	0.0022	0.0014	0.303 ± 0.073
24	BS, Clade A	*M. koshiensis *(6)	*M. pauciflora *(52)	1	0.0087	0.002	0.140 ± 0
25	BS, Clade A	*M. furusei *var.*subramosa *(24)	*M. pauciflora *(24)	6	0.0099	0.0034	0.343 ± 0.077
26	BS, Clade A	*M. furusei *var.*subramosa *(19)	*M. koshiensis *(5)	7	0.011	0.0027	0.271 ± 0.109
27	BS, Clade A	*M. acerina *(19)	*M. furusei *var.*subramosa *(19)	7	0.0122	0.0013	0.266 ± 0.124
28	BS, Clade A	*M. furusei *var.*subramosa *(19)	*M. acerina *(19)	7	0.0122	0.0013	0.254 ± 0.110
29	BS, Clade A	*M. furusei *var.*subramosa *(19)	*M. acerina *(19)	4	0.0122	0.0013	0.188 ± 0.021
30	BS, Clade A	*M. acerina *(19)	*M. furusei *var.*subramosa *(29)	3	0.0156	0.004	0.270 ± 0.173
31	BS, Clade B	*M. kiusiana *(60)	*M. stylosa *var. *makinoi *(71)	8	0.018	0.0068	0.274 ± 0.128
32	BS, Clade B	*M. kiusiana *(68)	*M. doiana *(72)	10	0.0203	0.0138	0.114 ± 0.097
33	BS, Clade B	*M. stylosa *var. *makinoi *(55)	*M. doiana *(72)	12	0.025	0.0054	0.068 ± 0.068
34	BS, Clade C	*M. yoshinagae *(69)	*M. japonica *(29)	11	0.0214	0	0.015 ± 0.019
35	BS, Clade C	*M. japonica *(60)	*M. yoshinagae *(47)	9	0.0301	0.0013	0.320 ± 0.238
36	BS, Clade C	*M. formosana *(77)	*M. japonica *(29)	3	0.0316	0.0075	0
37	BS, Clade C	*M. yoshinagae *(44)	*M. formosana *(77)	2	0.0316	0.0082	0.005 ± 0.007
38	BS, Clade C	*M. japonica *(60)	*M. yoshinagae *(64)	13	0.0354	0.0007	0.262 ± 0.136
39	BC	*M. acerina *(19)	M. *stylosa *var. *makinoi *(55)	2	0.0227	0.004	0.007 ± 0.007
40	BC	*M. furusei *var.*subramosa *(38)	*M. kiusiana *(67)	3	0.0288	0.0096	0.023 ± 0.040
41	BC	*M. koshiensis *(5)	*M. kiusiana *(60)	2	0.032	0.0075	0.030 ± 0.028
42	BC	*M. kiusiana *(68)	*M. pauciflora *(52)	6	0.0346	0.0089	0.028 ± 0.026
43	BC	*M. furusei *var.*subramosa *(24)	*M. stylosa *var. *makinoi *(55)	3	0.0347	0.0033	0.02
44	BC	*M. pauciflora *(52)	*M. stylosa *var. *makinoi *(45)	7	0.0371	0	0.103 ± 0.216
45	BC	*M. japonica *(60)	*M. acerina *(19)	4	0.0549	0.011	0
46	BC	*M. kiusiana *(60)	*M. japonica *(60)	3	0.0646	0.0007	0
47	BC	*M. japonica *(60)	*M. pauciflora *(39)	2	0.0672	0.0083	0
48	BC	*M. japonica *(60)	*M. furusei *var.*subramosa *(38)	3	0.0672	0.0103	0
49	BC	*M. formosana *(77)	*M. acerina *(19)	2	0.0691	0.0054	0
50	BC	*M. formosana *(77)	M. *stylosa *var. *makinoi *(55)	3	0.072	0.0027	0
51	BC	*M. yoshinagae *(64)	*M. kiusiana *(64)	3	0.0747	0.0013	0.003 ± 0.006
52	BC	*M. furusei *var.*subramosa *(29)	*M. japonica *(29)	2	0.0747	0.0082	0

a. Abbrebiations; wild: wild collected, cultivated plants examined for pollen fertility, WS: cross within biological species, BS: cross between species within the clade (A, B, C) defined in Figure 2, BC: cross across the clades.

b. Their geographic origins are shown as a number in parentheses, each of which corresponds to the population ID no. in Additional file 1: Table S1.

c. The number of plant individuals examined for their fertility.

**Figure 3:**
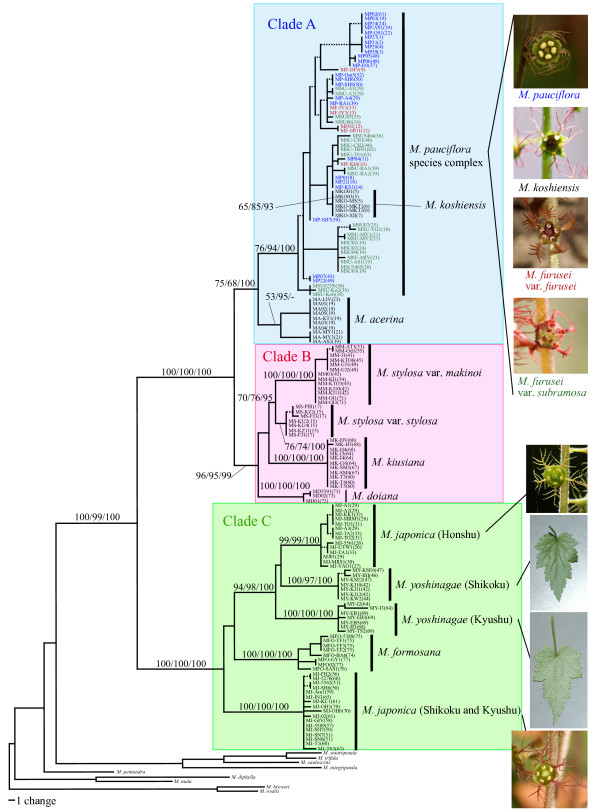
**One of the 8100 most parsimonious trees (L = 476, CI = 0.7542, RI = 0.9722) obtained by cladistic genotypic clustering of combined ETS and ITS sequences from 158 *Asimitellaria *plants and nine outgroups**. Branches that collapse in the strict consensus tree are shown by dashed lines. Nodal support values by bootstrapping or posterior probability are indicated near branches (MP/NJ/BI) where needed. Vertical bars on the right, with the exception of the *M. pauciflora *complex, represent distinct biological species proposed in the present study, which have substantial reproductive barriers to each other (> 39% of fertility reduction if crossed). For the *M. pauciflora *species complex, the labels in blue, red, and green each represent the taxonomic species *M. pauciflora, M. furusei *var. *furusei*, and * M. furusei *var. *subramosa*, respectively, at least among those that have a substantial level of reproductive isolation (> 57% of fertility reduction if crossed). Pictures on the right indicate some diagnostic characters (i.e., flower or abaxial side of the leaf) for (cryptic) biological species in several species complexes. The population ID nos. (see Additional file 1: Table S1 for details) from which the individual accessions were collected are indicated in parentheses.

Just prior to flowering, potted plants used for the crosses were transferred from the garden to growth chambers (NK System, Osaka, Japan) from which potential pollinator insects were excluded. Hermaphrodite flowers of maternal plants were emasculated before anthesis (this procedure was omitted for female flowers of several sexually dimorphic species), and subsequently used for the cross. A sufficient amount of pollen was applied onto the stigma of an emasculated flower using a toothpick. Three to 5 weeks after the cross, mature seeds were collected from these crossed fruits. The collected seeds were surface-sterilized for ~15 min with 0.04% TritonX-100 and sodium hypochlorite solution (~0.05% chlorine) and then plated onto sterilized nutrient agar in plastic Petri dishes. The resultant seeds germinated normally within 3 weeks of sowing. Two to 5 months after germination, the seedlings were transplanted to pots filled with well-fertilized soil and grown in the garden to examine their pollen viability in the next flowering season (March-May). Pollen grains were collected from each individual plant from just dehiscent anthers using a toothpick, and the pollen was scattered on a spot of liquid culture dropped onto a 1.5 cm × 1.5-cm square 1% agar culture, optimized for *Asimitellaria *pollen germination with 5% sucrose and 5.0 × 10^-3^% boric acid. After a 24-h incubation at 25°C in a humid plastic case, the agar culture with pollen grains was fixed with 3:1 ethanol: acetic acid, stained with 0.1% aniline blue for > 5 h, washed with 1% acetic acid, and dried to prepare microscope slides. For each slide specimen, 100 pollen grains were chosen at random to assess their germination ability under a binocular microscope, and the count was used as the measure of fertility for each individual plant. Accordingly, 1–19 individual measures of fertility were obtained for each of 43 intra- and interspecific cross strains. In addition, two to eight wild-collected individuals from each of nine *Asimitellaria *species were used to confirm their high pollen germination ability under our experimental conditions. To maximize the number of cross designs within limited time and space, we conducted the cross within a genotypic cluster as a control only in clade C, and instead used the measures of fertility for the wild-collected individuals originating presumably from spontaneous crosses within each genotypic cluster. The average pollen fertility was calculated for each of the 43 crossed strains and the nine wild-collected species. The artificially crossed strains within each of clades A-C observed in nuclear DNA phylogeny (Figure 3) with more than five individual plants were examined statistically for any reduction in hybrid fertility, compared to pollen fertility of corresponding wild-collected species using the Mann-Whitney U test with Bonferroni correction. The crossed strains across the clades were always nearly or completely sterile (Table 1); thus we did not assess these statistically for fertility reduction.

We further examined the correlations between genetic distance of parental plant individuals and their F1 hybrid fertility by plotting the results of the artificial cross experiments with parental nuclear or chloroplast genetic distances of the crossed lines. Genetic distance was simply calculated using the DNA sequences of parental individuals when available, whereas in several cases, a different plant individual of the same geographic origin was used as the representative of the accidentally lost plant used for the cross, as the intraspecific sequence divergence within a single population was always negligible (< 0.001 for chloroplast DNA and < 0.002 for nuclear DNA). For wild-collected plants and the intraspecific cross of plants from the same population (strain ID nos. 1–9, 15 in Table 1), their parental genetic distances were regarded as zero to discriminate them from the intraspecific cross of plants from different populations. A multiple regression analysis using a generalized additive model with smoothing splines was performed using each of the nuclear and chloroplast genetic distances as the independent variable and F1 hybrid pollen fertility as the dependent variable, applying the gam and smooth.spline function of the R package. An optimal regression model was selected by comparing the Akaike information criterion (AIC) values for all possible models, whereby nuclear and chloroplast genetic distance terms as well as their interaction term were incorporated in the full model.

## Results

The numbers of sites in the aligned data matrices of nuclear ribosomal ETS, ITS-1, ITS-2, and indels were 453, 276, 224, and 30, of which 84, 68, 47, and 24 were parsimony-informative, respectively, whereas those of *psbA-trnH*, *matK*, and indels (and an inversion) were 500, 1164, and 15, of which 28, 48, and 11 were parsimony-informative, respectively. Statistical summaries of genetic diversity observed within taxonomic species and varieties of *Asimitellaria *are listed in Table 2. Because chloroplast genetic distances in *Asimitellaria *were found to have very different information from that of the nuclear genetic distance, we regarded the nuclear and chloroplast DNA sequence data as different sources of information, and analyzed each separately.

**Table 2:** Statistical summaries of intraspecific genetic diversity of nuclear and chloroplast DNAs observed in *Asimitellaria*.

Species	No. of populations	No. of individuals	Nuclear distance mean (± S.D.)	Chloroplast distance mean (± S.D.)
*M. formosana*	4	8	0.0037 ± 0.0026	0.0005 ± 0.0004
*M. japonica *(all)	20	31	0.0185 ± 0.0171	0.0013 ± 0.0015
*M. japonica *(Honshu)	8	14	0.0010 ± 0.0013	0.0000 ± 0.0000
*M. japonica *(Shikoku and Kyushul)	12	17	0.0012 ± 0.0013	0.0021 ± 0.0014
*M. yoshinagae *(all)	7	14	0.0130 ± 0.0107	0.0011 ± 0.0011
*M. yoshinagae *(Shikoku)	4	7	0.0010 ± 0.0011	0.0021 ± 0.0013
*M. yoshinagae *(Kyushu)	3	7	0.0024 ± 0.0024	0.0000 ± 0.0000
*M. doiana*	2	3	0.0007 ± 0.0006	0.0004 ± 0.0004
*M. kiusiana*	4	11	0.0001 ± 0.0004	0.0012 ± 0.0010
*M. stylosa *(all)	11	20	0.0060 ± 0.0053	0.0016 ± 0.0014
*M. stylosa *var. *stylosa*	2	7	0.0005 ± 0.0007	0.0014 ± 0.0009
*M. stylosa *var. *makinoi*	9	13	0.0011 ± 0.0011	0.0001 ± 0.0002
*M. acerina*	3	10	0.0019 ± 0.0031	0.0007 ± 0.0007
*M. furusei *(all)	20	31	0.0079 ± 0.0044	0.0030 ± 0.0016
*M. furusei *var. *furusei*	4	6	0.0051 ± 0.0030	0.0025 ± 0.0013
*M. furusei *var. *subramosa*	16	25	0.0076 ± 0.0046	0.0030 ± 0.0017
*M. koshiensis*	3	6	0.0000 ± 0.0000	0.0016 ± 0.0014
*M. pauciflora*	20	24	0.0039 ± 0.0038	0.0015 ± 0.0012

Cladistic grouping of the nuclear ribosomal DNA sequences using MP, NJ, and BI all resulted in nearly identical topology, subdividing the entire ingroup into three subclades, A-C, in which two to five distinct genotypic clusters were consistently recognized (Figure 3). In total, at least 11 distinct subclades were found within *Asimitellaria *(Figure 3), and the identified clades were not largely incongruent with the present taxonomic system. *Mitella koshiensis*, *M. stylosa*, *M. kiusiana*, *M. doiana*, and *M. formosana *were always supported to be monophyletic, with moderate to high MP (65–100%) and NJ (76–100%) bootstrap values and high Bayesian posterior probability (> 93%). The monophyly of *M. acerina *was not supported by BI, although this was apparently due to genetic introgression of ITS sequences with sympatric *M. furusei *var. *subramosa*, which has been reported previously [20]. Intriguingly, as expected from the excess of their intraspecific genetic diversity (Table 2), none of the four taxonomic species, *M. pauciflora*, *M. furusei*, *M. japonica*, or *M. yoshinagae*, formed a monophyletic group, with the former two species forming an inseparable clade together with *M. koshiensis*, whereas *M. japonica *and *M. yoshinagae *were each composed of two non-sister clades with very high nodal support (> 97%; Figure 3). In addition, *M. stylosa *was further subdivided into two strongly supported (100%/100%/100% and 76%/74%/100% in MP, NJ, and BI, respectively) monophyletic clades, with each corresponding to the two taxonomic varieties, *M. stylosa *var. *stylosa *and *M. stylosa *var. *makinoi*.

**Figure 4:**
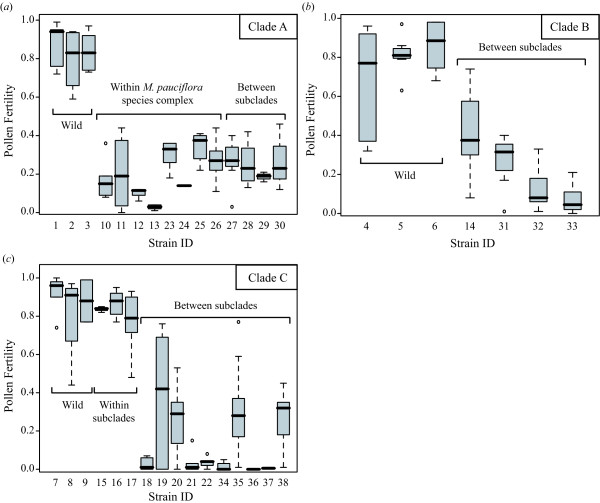
**Reduction in pollen fertility in artificially crossed interspecific hybrid strains of *Asimitellaria***. Boxes and error bars represent the range distribution for individual strains, with thick horizontal bars representing the median. (*a*) Crosses within clade A. (*b*) Crosses within clade B. (*c*) Crosses within clade C. The strain ID numbers are as in Table 1. Note that the strain IDs 1–9 are the wild-collected individuals used as controls.

The pairs of two distinct clades found within both *M. japonica *and *M. yoshinagae *have not been recognized previously, but careful reexamination of morphological characters found some support for these clusters from the morphology of petal and leaf surface structure (Figure 3). The measure of F1 hybrid sterility by artificial crossing experiments revealed the presence of clear reproductive barriers between the clusters. The cross between *M. japonica *individuals of the same genotypic cluster (Kyushu and Shikoku populations), each collected from geographically well isolated (279–335 km) populations, showed little hybrid sterility (Figure 4, Table 1, strain nos.16 and 17). Therefore, the significant reduction in pollen fertility in the F1 hybrids resulting from the inter-genotypic crosses cannot be explained by ordinary isolation by distance within a single species, whereby a gradual decrease in hybrid fertility is expected, but by an incompatibility between reproductively isolated, discontinuous species. Similarly, the two genotypic clusters observed in *M. stylosa*, corresponding to the taxonomic varieties *M. stylosa *var. *stylosa *and *M. stylosa *var. *makinoi*, would be two distinct species, as a significant reduction in hybrid fertility between them was observed. An exception is the species complex of *M. koshiensis*, *M. furusei*, and *M. pauciflora *(henceforth referred to as *M. pauciflora *complex), in which fairly large genetic variations were observed. Nevertheless, each of the three taxonomic species cannot be recognized as a distinct genotypic cluster.

**Figure 5:**
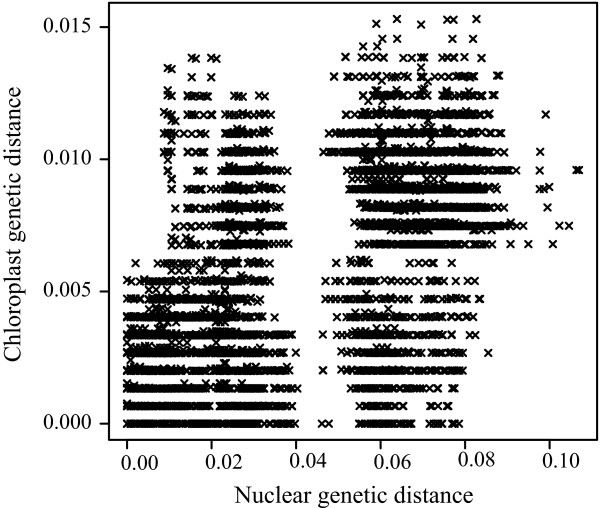
**Strong incongruence between nuclear and chloroplast genetic distance for all possible pairs of 141 *Asimitellaria *plant individuals**. The large gap along the x-axis (nuclear genetic distance) corresponds to the large genetic gap between clade A+B and clade C.

**Figure 6:**
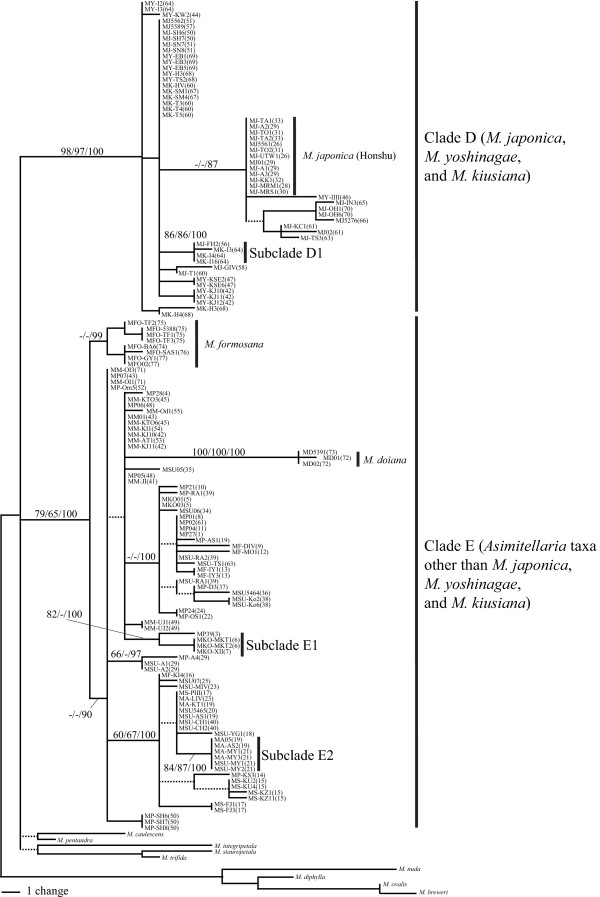
**One of the 7700 most parsimonious trees (L = 189, CI = 0.8042, RI = 0.9671) obtained via cladistic genotypic clustering of combined chloroplast DNA sequences from 141 *Asimitellaria *plants and nine outgroups**. D1, E1, and E2 are the strongly supported subclades, each consisting of two reproductively isolated, distinct biological species. Other descriptions are as in Figure 3.

**Figure 7:**
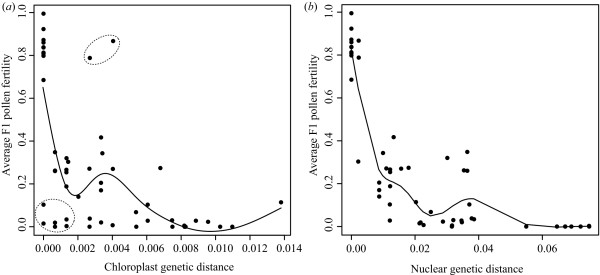
**Nonlinear, single regression of average F1 pollen fertility (*a*) against average parental chloroplast genetic distance (K81uf + G model of nucleotide substitutions with gamma shape = 0.2834) and (*b*) against the average parental nuclear genetic distance (Tamura--Nei + I + Γ model of nucleotide substitutions with gamma shape = 0.8578, proportion of invariable sites = 0.3018)**. Dashed circles indicate data points showing strong discordance between chloroplast genetic distance and average F1 pollen fertility.

Surprisingly, we found that the genetic information from chloroplast DNA sequences was very different from that from nuclear ETS and ITS DNAs in *Asimitellaria*. As shown in Figure 5, less nucleotide divergence in chloroplast DNA did not necessarily coincide with less divergence in ETS and ITS, and vice versa. In addition, unlike the nuclear ETS and ITS data, very few genotypic clusters that potentially correspond to species were found in chloroplast DNA. Actually, eight out of ten taxonomic species in *Asimitellaria *were recovered to be paraphyletic or polyphyletic in the chloroplast DNA data (e.g., subclades D1, E1, and E2 in Figure 6), although none of these groupings was supported morphologically. Only *M. formosana *and *M. doiana*, both of which are strictly allopatric with other *Asimitellaria *species, each formed an exclusively monophyletic group, although the nodal support for the *M. formosana *clade was weak (Figure 6). This pattern is consistent with our previous finding that chloroplast DNA in *Asimitellaria *is highly sensitive to rare interspecific gene flow [20]. This finding is further supported by the fact that the pattern of F1 hybrid pollen sterility expected from chloroplast genetic distances (Figure 7a; deviance explained = 52.8%) fitted the data much less compared to that from nuclear genetic distances (Figure 7b; deviance explained = 88.5%), and multiple regression analysis indicated that only the nuclear genetic distances are necessary and sufficient to explain the observed pattern of F1 hybrid pollen fertility (the AIC value for the optimal model was -64.04, whereas that for the full model was -63.62).

## Discussion

### The relationships among genetic divergence, postzygotic isolation, and taxonomic species boundaries in Asian *Mitella*, and their implications for DNA taxonomy

A rapidly evolving endeavor in recent taxonomy is to utilize DNA sequences for precise and efficient delineation of biodiversity [5], but the information regarding how observed genetic disparity corresponds to reproductive isolation has been critically lacking. In the present study, we have comprehensively illustrated the relationships among genetic divergence, postzygotic reproductive isolation, and taxonomic species boundaries using *Asimitellaria *as a model group. In *Asimitellaria*, we found that the degree of postzygotic reproductive isolation correlates consistently with genetic distance measured by nuclear ribosomal DNA only (Figure 7).

Consequently, the distinct subclades observed in the phylogeny (Figure 3) each corresponded to a distinct biological species, with the cross among which always result in at least 39% fertility reduction compared to the cross within the subclades (Table 1, Figure 4). Furthermore, we found that the genotypic clustering based on nuclear ribosomal DNA distance was mostly concordant with a morphology-based system (Figure 3). These findings have several significant implications for application of DNA taxonomy in flowering plants. Together with the previous finding in other three genera (*Glycine*, *Silene*, and *Strepthanthus *[16]), now we have a strong evidence to assume that in general the degree of postzygotic reproductive isolation well correlates with genetic distance (note here we assume no polyploidy, although it is undoubtedly a major factor generating reproductive isolation in plants). Nevertheless, the goodness of correlation can vary largely among genetic markers used for the distance measure, and thus careful examination is necessary to determine which marker should be chosen. In the marker choice for DNA taxonomy, comparing the relative goodness of fit to a morphology-based system might be very helpful; in the case of *Asimitellaria*, the marker that fit better to the pattern of reproductive isolation also fit better to the morphology-based system. Accordingly, in the case when artificial cross experiments are impractical, it would be a good practice for researchers to compare multiple, unlinked markers such as chloroplast DNA and nuclear ribosomal DNA for goodness of fit to the morphology-based system of the plant group under study.

We would note, however, that the postzygotic reproductive isolation measured by pollen fertility in the present study is only a very small fraction of reproductive isolation that exists in nature. It is suggested that the prezygotic isolation has more important role in keeping different species genetically distinct in both plants and animals [27]. Whether the degree of prezygotic reproductive isolation correlates consistently with genetic distance is not clear because prezygotic isolation involves many adaptive traits such as flowering time, pollinator difference, and floral morphology, which can be direct targets of natural selection [28]. Nevertheless, the general trend of strong correlations between genetic distance measured with a specific genetic marker and postzygotic reproductive isolation can be used (after the choice of appropriate markers) as a strong basis for regarding that phylogenetically supported distinct clusters can be used for a minimum assessment of biological species diversity.

Surprisingly, until now, limited examples of cryptic species within higher plants, including angiosperms, have been reported [29-32]. This may suggest that cryptic species are less frequent in flowering plants because the morphology-based taxonomic system is highly reliable; however, there have been too few empirical studies to make conclusive inferences regarding the prevalence of cryptic species within flowering plants. Nevertheless, considering the present findings of at least three cryptic species present within *Asimitellaria*, a relatively taxonomically well examined lineage with regard to comparative morphology, embryology, and cytology (e.g., [17,33,34]), it is likely that many angiosperm lineages contain at least some cryptic species (see also [30], who reported cryptic species diversity of the genus *Draba *in the Arctic region). Thus, the establishment of a concrete framework for DNA taxonomy would be valuable to unravel cryptic diversity of flowering plants.

### Utility and limitations of nuclear ribosomal DNA sequences for delimiting species

In the present study, genotypic clustering using nuclear ribosomal ETS and ITS sequences was shown to be fairly successful for delimiting biological species of *Asimitellaria *(ten of 11 clusters corresponded to distinct biological species), except for the *M. pauciflora *complex. This good correspondence was achieved from low intraspecific sequence diversity and consistent sequence monophyly for each biological species, in addition to the high overall variability of the sequences, an essential prerequisite for DNA taxonomy. It is noteworthy that recent plant DNA barcoding studies have suggested the potential utility of ITS regions for identifying plant taxonomic species, partly because it is the most frequently sequenced locus in plant phylogenetic studies. Importantly, Kress and colleagues found the highest interspecific sequence divergence of ITS among ten genetic markers tested [35], and Chase and colleagues reported a high probability (93.21%) of assigning taxonomic species using the ITS-1 region as a BLAST query sequence against GenBank [36]. However, in addition to these previously suggested advantages of ITS, it is especially important to achieve consistent species monophyly (each clade or genotypic cluster corresponding to one species) to be useful for species recognition and identification. Accordingly, the identifier loci are required to have a relatively short coalescence time. This may be the case in ETS and ITS regions because, unlike other nuclear loci, the sequence homogeneity within a genome is strongly maintained by concerted evolution [20,37,38]. Note that *Asimitellaria *is a paleotetraploid lineage (2n = 28), which has double chromosome number compared to most of the remaining species of *Mitella *and its allies (*Heuchera *group; mostly 2n = 14). Therefore, even the case in which ribosomal DNAs are likely located in multiple chromosome blocks, each of the species can be recognized consistently as a monophyletic group because of concerted evolution process comparable to those of diploids (see also [37,38], in which clear evidence for rapid concerted evolution among different chromosomal locations is reported).

Another ideal property of potential loci for plant species delimitation is their robustness for genetic introgression via interspecific hybridization, as plant species with morphological and/or ecological distinctiveness are often reported to have intensive gene exchange (e.g., [39]). It is theoretically predicted that the loci under divergent selection and those linked to hybrid incompatibility are unlikely to introgress across species [40], but it is usually very difficult to find such loci for many non-model plant lineages. The nuclear ribosomal gene is an exception, as in most eukaryotic genomes, including those of flowering plants, whereby the physical locations of highly repetitive, nuclear ribosomal gene clusters are confined within telomeric regions (*e.g*., [41,42]; the physical locations of ribosomal RNA genes for various model organisms are also available in the MapViewer at ) where recombination is presumably suppressed [43,44]. Importantly, in a recent genome-wide survey of genetic differentiation between frequently hybridizing, sympatric sunflower species, *Helianthus annuus *and *H. petiolaris*, Yatabe and colleagues found that the chromosomal segments that differentiated these species are usually very small (even undetectable in sunflowers) except for the regions neighboring chromosomal breakpoints [39]. Consequently, it is possible that the ribosomal ETS and ITS sequences, which are located near the chromosomal breakpoints, remain distinct under a substantial degree of interspecific genetic introgression, as in the case of *Asimitellaria *[20].

Taken together, we propose that the ETS and ITS regions are the most promising currently available candidate markers for DNA taxonomy of flowering plants, with presumably short coalescent times and robustness against introgressive hybridization [20]; note, however, that there have been several reports of interspecific gene flow in the ITS regions [45,46]. Even in *Asimitellaria*, there is a clear example of interspecific gene flow in ITS region (but not in ETS) between *M. acerina *and *M. furuei *var. *subramosa *[20]. Therefore, it is worth testing in *Asimitellaria *and other plant lineages whether nuclear ribosomal DNA is indeed more robust against interspecific gene flow compared to other loci such as protein-coding nuclear genes. Of course, in either case, it would be better to keep in mind that there is unlikely to be any genetic markers free of interspecific gene flow, which is always a major challenge in plant taxonomy.

Moreover, in some cases, another caution is needed for use of the ETS and ITS because there are non-negligible numbers of reports for the presence of multiple divergent paralogs of ribosomal DNA in a single genome [47-49], which makes it impossible to compare orthologous sequences among individuals, an essential step for DNA taxonomy. Such a problematic nature of ribosomal DNA for plant DNA taxonomy might be more general phenomenon, considering even reporting bias might be present for the plant group in which ribosomal DNAs cannot be sequenced directly in a simple manner as in *Asimitellaria*.

Also note that even our genotypic clustering approach failed to recognize the three taxonomic species within the *M. pauciflora *complex (Figure 3), especially to discriminate between *M. furusei *and *M. pauciflora *(*M. koshiensis *could be recognized with a species-specific sequence nested within the complex in the phylogenetic tree). This does not mean that the complex should be grouped as a single biological species, as the phenotypic discontinuity among the three taxonomic species is obvious ([17,18]; Figure 3), and *M. furusei *and *M. pauciflora *co-occur in many populations and have very distinct life history traits, including flowering phenology, pollinator species, and modes of reproduction [19,50]. In addition, the artificial cross experiments within the *M. pauciflora *complex (Figure 4) also supported the conclusion that the three taxonomic species are reproductively isolated. Even the cross between genetically divergent populations of *M. furusei *var. *subramosa *was found to result in strong hybrid sterility (< 10% pollen fertility; strain ID nos. 12 and 13 in Figure 4). Therefore, it is clear that the species complex comprises more than three biological species, although clarifying a comprehensive pattern of reproductive isolation within the *M. pauciflora *complex is beyond the scope of the present study. The divergence between *M. furusei *and *M. pauciflora *appears to have occurred very recently compared to other speciation events outside the *M. pauciflora *complex, and this observation probably indicates limitation to the sole use of ETS and ITS sequences for recognizing plant biological species that have undergone recent speciation. Accordingly, it is expected that a recently radiated plant lineage would be most difficult for biological species recognition based on ETS and ITS sequences, even if sequence variations are present, as in the case of the *M. pauciflora *complex. At present, there is no conventional, DNA-based methodology for recognizing these recently diverged biological species (amplified fragment length polymorphism-PCR may be a candidate, although this method is fairly labor-intensive; e.g., [51,52]). A recent simulation-based study suggested that sampling of a moderate number (up to ten) of loci could correctly delimit recently diverged biological species with a coalescent theory-based approach, even without relying on their reciprocal monophyly [53]. Thus, there is no doubt that adding the data from different loci would result in more precise delimitation of biological species, including those that have differentiated recently, although the cost and effort would also increase substantially.

## Conclusion

To our knowledge, this is the first comprehensive study that links quantitative measures of postzygotic reproductive isolation to genetic distinctness observed in an angiosperm lineage. We showed that with appropriate selection of genetic markers, most reproductively isolated species of *Asimitellaria *could be recognized as distinct genotypic clusters. With only the present results being available, however, we could not conclude whether the low efficiency of biological species recognition using chloroplast DNA is a general trend in flowering plants. It is widely appreciated that chloroplast DNA has a general advantage of the availability of nearly universal primers that are applicable to entire flowering plants, and less risk of sampling multiple copies from one plant individual, which in turn is one of the major obstacles in using nuclear ribosomal DNA sequences [47-49]. Nevertheless, extensive introgression of chloroplast DNA via interspecific hybridization is a well-known and frequently reported phenomenon not restricted to *Asimitellaria *(*e.g*., [54-57]; older examples are reviewed in [58]). Therefore, it would be necessary to reassess how common chloroplast DNA introgression is among flowering plants, especially after sampling a sufficient number of individuals for each taxonomic or biological species. Also, it might be helpful to compare the use of nuclear ribosomal and chloroplast DNA in plant lineages without chloroplast DNA introgression. Further attempts at DNA taxonomy in plant lineages with various life history traits (annuals, perennials, trees, aquatics) and evolutionary backgrounds (recent and old radiations, oceanic island endemics) are required to generalize the utility of ETS and ITS for accurate and efficient delimitation of plant biological species.

## Authors' contributions

YO designed and performed the research, analyzed the data, and wrote the manuscript. MK designed research and wrote the manuscript. Both authors read and approved the final manuscript.

## Supplementary Material

Additional file 1 **Tables S1 and S2**. Table S1: Population localities from which ETS and ITS sequences were obtained. The species name (with its acronym for figures 3 and 6 in parentheses) and the number of individuals sampled for each population are also indicated. Table S2: Primer sequences used in the study. Click here for file

Additional file 2 **Additional text**. Methods for DNA extraction, sequencing, and data preparation. Click here for file

## References

[B1] Savolainen V, Chase MW (2003). A decade of progress in plant molecular phylogenetics. Trends Genet.

[B2] The Angiosperm Phylogeny Group (1998). An ordinal classification for the families of flowering plants. Ann MO Bot Gard.

[B3] The Angiosperm Phylogeny Group (2003). An update of the Angiosperm Phylogeny Group classification for the orders and families of flowering plants: APG II. Bot J Linn Soc.

[B4] Monaghan MT, Balke M, Pons J, Vogler AP (2006). Beyond barcodes: complex DNA taxonomy of a South Pacific Island radiation. Proc R Soc B.

[B5] Vogler AP, Monaghan MT (2007). Recent advances in DNA taxonomy. J Zool Syst Evol Res.

[B6] Syring J, Farrell K, Businsky R, Cronn R, Liston A (2007). Widespread genealogical nonmonophyly in species of *Pinus *subgenus *Strobus*. Syst Biol.

[B7] Levin DA (1979). The nature of plant species. Science.

[B8] Diamond JM (1992). Horrible plant species. Nature.

[B9] Mayr E (1992). A local flora and the biological species concept. Am J Bot.

[B10] Rieseberg LH, Wood TE, Baack EJ (2006). The nature of plant species. Nature.

[B11] Rieseberg LH, Brouillet L (1994). Are many plant species paraphyletic?. Taxon.

[B12] Chase MW, Cowan RS, Hollingsworth PM, Berg C van den, Madriñán S, Petersen G, Seberg O, Jørgsensen T, Cameron KM, Carine M, Pedersen N, Hedderson TAJ, Conrad F, Salazar GA, Richardson JE, Hollingsworth ML, Barraclough TG, Kelly L, Wilkinson M (2007). A proposal for a standardised protocol to barcode all land plants. Taxon.

[B13] Kress WJ, Erickson DL (2007). A Two-locus global DNA barcode for land plants: the coding *rbcL *gene complements the non-coding *trnH-psbA *spacer region. PLoS One.

[B14] Taberlet P, Coissac E, Pompanon F, Gielly L, Miquel1 C, Valentini1 A, Vermat T, Corthier G, Brochmann C, Willerslev E (2007). Power and limitations of the chloroplast *trnL *(UAA) intron for plant DNA barcoding. Nucl Acids Res.

[B15] Lahaye R, Bank M van der, Bogarin D, Warner J, Populin F, Gigot G, Maurin O, Duthoit S, Barraclough TG, Savolainen V (2008). DNA barcoding the floras of biodiversity hotspots. Proc Natl Acad Sci USA.

[B16] Moyle LC, Olson MS, Tiffin P (2004). Patterns of reproductive isolation in three angiosperm genera. Evolution.

[B17] Wakabayashi M (1973). A note on the genus *Mitella *of Japan. Acta Phytotax Geobot.

[B18] Wakabayashi M, Iwatsuki K, Boufford DE, Ohba H (2001). Saxifragaceae 13. *Mitella*. Flora of Japan.

[B19] Okuyama Y, Pellmyr O, Kato M (2008). Parallel floral adaptations to pollination by fungus gnats within the genus *Mitella *(Saxifragaceae). Mol Phylogenet Evol.

[B20] Okuyama Y, Fujii N, Wakabayashi M, Kawakita A, Ito M, Watanabe M, Murakami N, Kato M (2005). Nonuniform concerted evolution and chloroplast capture: heterogeneity of observed introgression patterns in three molecular data partition phylogenies of Asian *Mitella *(Saxifragaceae). Mol Biol Evol.

[B21] Simmons MP, Ochoterena H (2000). Gaps as characters in sequence-based phylogenetic analyses. Syst Biol.

[B22] Swofford DL (2002). PAUP*, Phylogenetic Analysis Using Parsimony (*and Other Methods). Version 4.

[B23] Posada D, Crandall KA (1998). MODELTEST: testing the model of DNA substitution. Bioinformatics.

[B24] R Development Core Team (2008). R: A language and environment for statistical computing.

[B25] Huelsenbeck JP, Ronquist F (2001). MRBAYES: Bayesian inference of phylogenetic trees. Bioinformatics.

[B26] Nylander JAA (2004). MrModeltest v2.

[B27] Lowry DB, Modliszewski JL, Wright KM, Wu CA, Willis JH (2008). The strength and genetic basis of reproductive isolating barriers in flowering plants. Phil Trans R Soc B.

[B28] Coyne JA, Orr HA (2004). Speciation.

[B29] Bickford D, Lohman DJ, Sodhi NS, Ng PKL, Meier R, Winker K, Ingram KK, Das I (2007). Cryptic species as a window on diversity and conservation. Trends Ecol Evol.

[B30] Grundt HH, Kjølner S, Borgen L, Rieseberg LH, Brochmann C (2006). High biological species diversity in the arctic flora. Proc Natl Acad Sci USA.

[B31] Whittall JB, Hellquist CB, Schneider EL, Hodges SA (2004). Cryptic species in an endangered pondweed community (*Potamogeton*, Potamogetonaceae) revealed by AFLP markers. Am J Bot.

[B32] Nicolè F, Tellier F, Vivat A, Till-Bottraud I (2007). Conservation unit status inferred for plants by combining interspecific crosses and AFLP. Conserv Genet.

[B33] Wakabayashi M (1977). A note on *Mitella stylosa *and allied species (Saxifragaceae). Acta Phytotax Geobot.

[B34] Wakabayashi M (1992). Embryology of Japanese *Mitella *(Saxifragaceae) and its taxonomic significance. Bot Mag Tokyo.

[B35] Kress WJ, Wurdack KJ, Zimmer EA, Weigt LA, Janzen DH (2005). Use of DNA barcodes to identify flowering plants. Proc Natl Acad Sci USA.

[B36] Chase MW, Salamin N, Wilkinson M, Dunwell JM, Kesanakurthi RP, Haidar N, Savolainen V (2005). Land plants and DNA barcodes: short-term and long-term goals. Phil Trans R Soc B.

[B37] Wendel JF, Schnabel A, Seelanan T (1995). Bidirectional interlocus concerted evolution following allopolyploid speciation in cotton (*Gossypium*). Proc Natl Acad Sci USA.

[B38] Soltis DE, Soltis PS, Pires JC, Kovarik A, Tate JA, Mavrodiev E (2004). Recent and recurrent polyploidy in *Tragopogon *(Asteraceae): cytogenetic, genomic and genetic comparisons. Biol J Linn Soc.

[B39] Yatabe Y, Kane NC, Scotti-Saintagne C, Rieseberg LH (2007). Rampant gene exchange across a strong reproductive barrier between the annual sunflowers, *Helianthus annuus *and *H. petiolaris*. Genetics.

[B40] Barton NH (1979). Dynamics of hybrid zones. Heredity.

[B41] Zhang D, Sang T (1999). Physical mapping of ribosomal RNA genes in peonies (*Paeonia*, Paeoniaceae) by fluorescent in situ hybridization: implications for phylogeny and concerted evolution. Am J Bot.

[B42] Shishido R, Sano Y, Fukui K (2000). Ribosomal DNAs: an exception to the conservation of gene order in rice genomes. Mol Gen Genet.

[B43] Gerton JL, DeRisi J, Shroff R, Lichten M, Brown PO, Petes TD (2000). Global mapping of meiotic recombination hotspots and coldspots in the yeast *Saccharomyces cerevisiae*. Proc Natl Acad Sci USA.

[B44] Wu JZ, Mizuno H, Hayashi-Tsugane M, Ito Y, Chiden Y, Fujisawa M, Katagiri S, Saji S, Yoshiki S, Karasawa W, Yoshihara R, Hayashi A, Kobayashi H, Ito K, Hamada M, Okamoto M, Ikeno M, Ichikawa Y, Katayose Y, Yano M, Matsumoto T, Sasaki T (2003). Physical maps and recombination frequency of six rice chromosomes. Plant J.

[B45] Aguilar JF, Rosselló JA, Feliner GN (1999). Molecular evidence for the compilospecies model of reticulate evolution in *Armeria *(Plumbaginaceae). Syst Biol.

[B46] Aguilar JF, Rosselló JA, Feliner GN (1999). Nuclear ribosomal DNA (nrDNA) concerted evolution in natural and artificial hybrids of *Armeria *(Plumbaginaceae). Mol Ecol.

[B47] Buckler-IV ES, Ippolito A, Holtsford TP (1997). The evolution of ribosomal DNA: divergent paralogues and phylogenetic implications. Genetics.

[B48] Alvarez I, Wendel JF (2003). Ribosomal ITS sequences and plant phylogenetic inference. Mol Phylogenet Evol.

[B49] Bailey CD, Carr TG, Harris SA, Hughes CE (2003). Characterization of angiosperm nrDNA polymorphism, paralogy, and pseudogenes. Mol Phylogenet Evol.

[B50] Okuyama Y, Kato M, Murakami N (2004). Pollination by fungus gnats in four species of the genus *Mitella *(Saxifragaceae). Bot J Linn Soc.

[B51] Kardolus JP, van Eck HJ, Berg RG van den (1998). The potential of AFLPs in biosystematics: A first application in *Solanum *taxonomy (Solanaceae). Plant Syst Evol.

[B52] Pellmyr OP, Segraves KA, Althoff DM, Balcázar-Lara M, Leebens-Mack J (2007). The phylogeny of yuccas. Mol Phylgenet Evol.

[B53] Knowles LL, Carstens BC (2007). Delimiting species without monophyletic gene trees. Syst Biol.

[B54] Kron KA, Gawen LM, Chase MW (1993). Evidence for introgression in azaleas (*Rhododendron*; Ericaceae): chloroplast DNA and morphological variation in a hybrid swarm on Stone Mountain, Georgia. Am J Bot.

[B55] Petit RJ, Pineau E, Demesure B, Bacilieri R, Ducousso A, Kremer A (1997). Chloroplast DNA footprints of postglacial recolonization by oaks. Proc Natl Acad Sci USA.

[B56] Bänfer G, Moog U, Fiala B, Mohamed M, Weising K, Blattner FR (2006). A chloroplast genealogy of myrmecophytic *Macaranga *species (Euphorbiaceae) in Southeast Asia reveals hybridization, vicariance and long-distance dispersals. Mol Ecol.

[B57] Fehrer J, Gemeinholzerb B, Chrtek J, Bräutigam S (2007). Incongruent plastid and nuclear DNA phylogenies reveal ancient intergeneric hybridization in *Pilosella *hawkweeds (*Hieracium*, Cichorieae, Asteraceae). Mol Phylogenet Evol.

[B58] Rieseberg LH, Soltis DE (1991). Phylogenetic consequences of cytoplasmic gene flow in plants. Evol Trend Plant.

