# Supplementary material for: Unveiling cryptic species diversity of flowering plants: successful biological species identification of Asian Mitella using nuclear ribosomal DNA sequences
Source: BMC Evol Biol. 2009 May 16;9:105. doi: 10.1186/1471-2148-9-105 (PMC2695457; doi:10.1186/1471-2148-9-105)
Supplement: Additional file 1 — Tables S1 and S2. Table S1: Population localities from which ETS and ITS sequences were obtained. The species name (with its acronym for figures 3 and 6 in parentheses) and the number of individuals sampled for each population are also indicated. Table S2: Primer sequences used in the study. [file 1471-2148-9-105-S1.doc]

| Table S1. Population localities from which ETS and ITS sequences were obtained. The species name (with its acronym for figures 3 and 6 in parentheses) and the number of individuals sampled for each population are also indicated. | | | |
| --- | --- | --- | --- |
| Sampled species | Population ID | Locality | No. of individuals |
| *M. acerina* (MA) | 19 | Ashu, Miyama-cho, Kyoto pref. | 7 |
|  | 21 | Yohoro, Maizuru-city, Kyoto pref. | 2 |
|  | 23 | Kodeishi, Kyoto city, Kyoto pref. | 1 |
| *M. doiana* (MD) | 72 | Shiratani, Yakushima-cho, Yakushima Isl., Kagoshima pref. | 1 |
|  | 73 | Akatsuka, Yakushima-cho, Yakushima Isl., Kagoshima pref. | 2 |
| *M. formosana* (MFO) | 74 | Pouluoshan, Datong Hsiang, Ylan Co., Taiwan | 1 |
|  | 75 | Tsuei Feng, Jenai Hsiang, Nantou Co., Taiwan | 4 |
|  | 76 | Shang-lin-xi, Zhushan Zhen, Nantou Co., Taiwan | 1 |
|  | 77 | Mt. Lulinshan, Alishan Hsiang, Nantou Co., Taiwan | 2 |
| *M. furusei* var. *furusei* (MF) | 9 | Yamaguchi-mura, Nagano pref. | 1 |
|  | 12 | Misakubo-cho, Shizuoka pref. | 2 |
|  | 13 | Ikenouchi, Inuyama city, Aichi pref. | 2 |
|  | 16 | Ibi-kyo, Ibigaya-cho, Gifu pref. | 1 |
| *M. furusei* var. *subramosa* (MSU) | 18 | Yogo-cho, Shiga pref. | 1 |
|  | 19 | Ashu, Miyama-cho, Kyoto pref. | 4 |
|  | 20 | Kasahara, Takahama-cho, Fukui pref. | 1 |
|  | 21 | Yohoro, Maizuru-city, Kyoto pref. | 2 |
|  | 23 | Kodeishi, Kyoto city, Kyoto pref. | 1 |
|  | 24 | Kibune Okuno-in, Kyoto city, Kyoto pref. | 1 |
|  | 25 | Mt. Daimonji, Kyoto city, Kyoto pref. | 1 |
|  | 29 | Akame 48 falls, Nabari-city, Mie pref. | 2 |
|  | 34 | Takanogawa, Nachi-katsuura-cho, Wakayama pref. | 1 |
|  | 35 | Kusukawa, Nachi-katsuura-cho, Wakayama pref. | 1 |
|  | 36 | Mt. Kyusho, Tottori city, Tottori pref. | 1 |
|  | 38 | Koryo-cho, Shimane pref. | 3 |
|  | 39 | Rakan-keikoku, Hatsukaichi city, Hiroshima pref. | 2 |
|  | 40 | Chomonkyo, Ato-cho, Yamaguchi pref. | 2 |
|  | 62 | Nakayama, Tara-cho, Saga pref. | 1 |
|  | 63 | Todoroki-kyo, Takaki-cho, Nagasaki pef. | 1 |
| *M. japonica* (MJ) | 26 | Mt. Taiho, Uji-tawara-cho, Kyoto pref. | 2 |
|  | 27 | Kodachi, Yao city, Osaka pref. | 1 |
|  | 28 | Mukobuchi, Murou-mura, Nara pref. | 1 |
|  | 29 | Akame 48 falls, Nabari-city, Mie pref. | 4 |
|  | 30 | Shimodaguchi, Murou-mura, Nara pref. | 1 |
|  | 31 | Mt. Tonomine, Kashihara city, Nara pref. | 2 |
|  | 32 | Shimotako, Kawakami-mura, Nara pref. | 1 |
|  | 33 | Abu-touge, Tenkawa-mura, Nara pref. | 2 |
|  | 50 | Higashinokawa-dani, Saijo-city, Ehime pref. | 2 |
|  | 51 | Saijo city, Ehime pref. | 3 |
|  | 56 | Hiraodai, Kitakyushu city, Fukuoka pref. | 1 |
|  | 57 | Mt. Hikosan, Soeda-cho, Fukuoka pref. | 1 |
|  | 58 | Kyusuikei, Kokonoe-cho, Oita pref. | 1 |
|  | 59 | Amano-iwato, Takachiho-cho, Miyazaki pref. | 1 |
|  | 60 | Takachiho-kyo, Takachiho-cho, Miyazaki pref. | 1 |
|  | 61 | Kikuchi-keikoku, Kikuchi city, Kumamoto pref. | 2 |
|  | 63 | Todoroki-kyo, Takaki-cho, Nagasaki pef. | 1 |
|  | 65 | Mt. Noke-eboshi, Itsuki-mura, Kumamoto pref. | 1 |
|  | 66 | Tsutsumi, Kuma-mura, Kumamoto pref. | 1 |
|  | 70 | Mt. Hoyoshi-dake, Uchinoura-cho, Kagoshima pref. | 2 |
| *M. kiusiana* (MK) | 60 | Takachiho-kyo, Takachiho-cho, Miyazaki pref. | 4 |
|  | 64 | Otaki, Itsuki-mura, Kumamoto pref. | 3 |
|  | 67 | Samukawa, Saito city Miyazaki pref. | 2 |
|  | 68 | Horikiri-dani, Taragi-cho, Kumamoto pref. | 2 |
| *M. koshiensis* (MKO) | 5 | Numagoshi-toge, Mikawa-mura, Niigata pref. | 3 |
|  | 6 | Mt. Kakuda-yama, Nigata city, Niigata pref. | 2 |
|  | 7 | Miyazaki, Asahi-cho, Toyama pref. | 1 |
| *M. pauciflora* (MP) | 1 | Chokei-daira, Fukaura-cho, Aomori pref. | 1 |
|  | 2 | Otaki, Oga city, Akita pref., | 1 |
|  | 3 | Dakigaeri-keikoku, Tazawako-cho, Akita pref. | 1 |
|  | 4 | Mt. Taihaku, Sendai City, Miyagi pref. | 1 |
|  | 8 | Tochimoto, Kiso-fukushima city, Nagano pref. | 1 |
|  | 10 | Mt. Hakusan, Shiramine-mura, Ishikawa pref. | 1 |
|  | 11 | Hakone-cho, Kanagawa pref. | 1 |
|  | 14 | Shibakura-dani, Kuze-mura, Gifu pref. | 1 |
|  | 19 | Ashu, Miyama-cho, Kyoto pref. | 2 |
|  | 22 | Shirutani, Otsu city, Shiga pref. | 1 |
|  | 24 | Kibune Okuno-in, Kyoto city, Kyoto pref. | 1 |
|  | 29 | Akame 48 falls, Nabari-city, Mie pref. | 1 |
|  | 37 | Daisendera, Daisen-cho, Tottori pref. | 1 |
|  | 39 | Rakan-keikoku, Hatsukaichi city, Hiroshima pref. | 1 |
|  | 43 | Omiya-dani, Ikeda-cho, Toikushima pref. | 1 |
|  | 48 | Mt. Tsurugi, Miyoshi city, Tokushima pref. | 2 |
|  | 49 | Yanaze, Umaji-mura, Kochi pref. | 1 |
|  | 50 | Higashinokawa-dani, Saijo-city, Ehime pref. | 3 |
|  | 52 | Omogo-kei, Kumakogen-cho, Ehime pref. | 1 |
|  | 61 | Kikuchi-keikoku, Kikuchi city, Kumamoto pref. | 1 |
| *M. stylosa* var. *stylosa* (MS) | 15 | Kosaka, Kuze-mura, Gifu pref. | 4 |
|  | 17 | Mt. Fujiwara-dake, Inabe city, Mie pref. | 3 |
| *M. stylosa* var. *makinoi* (MM) | 41 | Mt. Koetsu-yama, Yamakawa-cho, Tokushima pref. | 1 |
|  | 42 | Kanjo-taki, Sade, Mima city, Tokushima pref. | 2 |
|  | 43 | Omiya-dani, Ikeda-cho, Toikushima pref. | 1 |
|  | 45 | Umikawa, Kito-mura, Tokushima pref. | 2 |
|  | 49 | Yanaze, Umaji-mura, Kochi pref. | 2 |
|  | 53 | Teramura, Agawa-cho, Kochi pref. | 1 |
|  | 54 | Irazu-keikoku, Tsuno-cho, Kochi pref. | 1 |
|  | 55 | Oda-miyama, Oda-cho, Ehime pref. | 1 |
|  | 71 | Mt. Inao-dake, Sata-cho, Kagoshima pref. | 2 |
| *M. yoshinagae* (MY) | 42 | Kanjo-taki, Sade, Mima city, Tokushima pref. | 3 |
|  | 44 | Kisawa-mura, Tokushima pref. | 1 |
|  | 46 | Karei-dani, Kainan-cho, Tokushima pref. | 1 |
|  | 47 | Semidani, Naka-cho, Tokushima pref. | 2 |
|  | 64 | Otaki, Itsuki-mura, Kumamoto pref. | 2 |
|  | 68 | Horikiri-dani, Taragi-cho, Kumamoto pref. | 2 |
|  | 69 | Kuruson shrine, Ebino city, Miyazaki pref. | 3 |

| Table S2. Primer sequences used in the study | |  |
| --- | --- | --- |
| Primer name | Sequences | Locus |
| F-ETS1 *Heu* | GGTGCCTAAAATGCGTGGGTGGACAGG | ETS |
| 18S-IGS | GAGACAAGCATATGACTACTGGCAGGATCAACCAG | ETS (18S rDNA) |
| 18S-E | GCAGGATCAACCAGGTGACA | ETS (18SrDNA) |
| Nnc18S10 | AGGAGAAGTCGTAACAAG | ITS (18SrDNA) |
| C26A | GTTTCTTTTCCTCCGCT | ITS (26SrDNA) |
| 307R | TTGGGCTGCATTCCCA | ITS (26SrDNA) |
| psbA3'f | GTTATGCATGAACGTAATGCTC | *psbA-trnH* (*psbA*) |
| trnHf | CGCGCATGGTGGATTCACAATCC | *psbA-trnH* (*trnH*) |
| Angio-trnKf | GAACCCGGAACTAGTCGGATG | *matK* (*trnK*) |
| Angio-trnKr | AAGACCACGACTGATCCT | *trnK* (*trnK*) |
| *Heu*-matK3268F | TATTTATGCACTTGCTCATGAT | *matK* |
| *Heu*-matK-F523 | CTTATGTATGTGAATACGAATC | *matK* |
| *Heu*-matK-R601 | GTTCCAGAAGATGTTGATCG | *matK* |
| *Heu*-matK-R1 | CGAGCACAAGAAAGTCGAAG | *matK* |
