# Supplementary material for: Unveiling cryptic species diversity of flowering plants: successful biological species identification of Asian Mitella using nuclear ribosomal DNA sequences
Source: BMC Evol Biol. 2009 May 16;9:105. doi: 10.1186/1471-2148-9-105 (PMC2695457; doi:10.1186/1471-2148-9-105)
Supplement: Additional file 2 — Additional text. Methods for DNA extraction, sequencing, and data preparation. [file 1471-2148-9-105-S2.doc]

**Methods for DNA extraction, sequencing, and data preparation**

Silica-dried or fresh leaves from cultivated plants or herbarium specimens were ground in liquid nitrogen with a mortar and pestle, washed with HEPES buffer (pH 8.0), and subjected to standard hexadecyltrimethylammonium bromide (CTAB) extraction to obtain clean total genomic DNA. For most plant materials, the contiguous arrays of ETS-18S-ITS ribosomal DNA (ca. 2.5 kb) were amplified by polymerase chain reaction (PCR) using the forward primer F-ETS1 *Heu* and reverse primer 307R. The chloroplast *psbA-trnH* spacer region was always amplified using the forward primer psbA3’f and reverse primer trnHf following [36]. *MatK* gene DNA sequences, which cover the entire 774bp region commonly used for plant DNA barcoding [16], were amplified using either of the primer pairs Angio-trnKf and Angio-trnKr or *Heu*-matK3268F and *Heu*-matK-R1. For somewhat degraded DNA materials, mostly from herbarium specimens, ETS and ITS regions were separately PCR amplified with the primer sets F-ETS1 *Heu* and 18S-IGS and Nnc18S10 and C26A, respectively. Likewise, the 5’ and 3’ halves of the *matK* gene were separately amplified with the primer sets *Heu*-matK3268F and *Heu*-matK-R601 and *Heu*-matK-F523 and *Heu*-matK-R1, respectively. PCR products were purified by polyethylene glycol precipitation, and used for sequencing with an ABI Big Dye Terminator Cycle Sequence Ready kit ver. 3.0 and ABI 3100 or 3130 DNA Sequencer (Applied Biosystems, Foster City, CA) using the following primers, F-ETS1 *Heu*, 18S-E, Nnc18S10, C26A, psbA3’f, trnHf, *Heu*-matK3268F, *Heu*-matK-R601, *Heu*-matK-F523, and *Heu*-matK-R1. The DNA sequences generated in previous studies [19,20], were also compiled within the dataset.
